# Fault-tolerant error correction with the gauge color code

**DOI:** 10.1038/ncomms12302

**Published:** 2016-07-29

**Authors:** Benjamin J. Brown, Naomi H. Nickerson, Dan E. Browne

**Affiliations:** 1 Niels Bohr International Academy, Niels Bohr Institute, Blegdamsvej 17, 2100 Copenhagen, Denmark; 2 Quantum Optics and Laser Science, Blackett Laboratory, Imperial College London, Prince Consort Road, London SW7 2AZ, UK; 3 Department of Physics and Astronomy, University College London, Gower Street, London WC1E 6BT, UK

## Abstract

The constituent parts of a quantum computer are inherently vulnerable to errors. To this end, we have developed quantum error-correcting codes to protect quantum information from noise. However, discovering codes that are capable of a universal set of computational operations with the minimal cost in quantum resources remains an important and ongoing challenge. One proposal of significant recent interest is the gauge color code. Notably, this code may offer a reduced resource cost over other well-studied fault-tolerant architectures by using a new method, known as gauge fixing, for performing the non-Clifford operations that are essential for universal quantum computation. Here we examine the gauge color code when it is subject to noise. Specifically, we make use of single-shot error correction to develop a simple decoding algorithm for the gauge color code, and we numerically analyse its performance. Remarkably, we find threshold error rates comparable to those of other leading proposals. Our results thus provide the first steps of a comparative study between the gauge color code and other promising computational architectures.

## Abstract (web summary)

Construction of a scalable quantum computer requires error-correcting codes to overcome the errors introduced by noise. Here, the authors develop a decoding algorithm for the gauge color code, and obtain its threshold values when physical errors and measurement faults are included.

## Graphical abstract

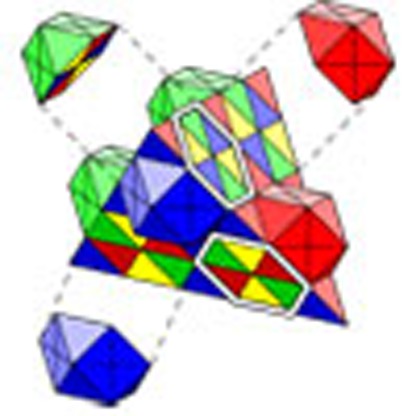

Scalable quantum technologies require the ability to maintain and manipulate coherent quantum states over an arbitrarily long period of time. It is problematic then that the small quantum systems that we might use to realize such technologies decohere rapidly due to unavoidable interactions with the environment. To resolve this issue we have discovered quantum error-correcting codes^1^,^2^, which make use of a redundancy of physical qubits to maintain encoded quantum states with arbitrarily high fidelity over an indefinite period.

Ideally, we will design a fault-tolerant quantum computer that requires as few physical qubits as possible to minimize the resource cost of a quantum processor and, indeed, the cost in resources of a computational architecture is very sensitive to the choice of quantum error-correcting code used by a fault-tolerant scheme. It is therefore of great interest to analyse different quantum error-correction proposals, to compare and contrast their resource demands.

Color codes^3^,^4^,^5^,^6^,^7^ are a family of topological quantum error-correcting codes^8^,^9^,^10^,^11^ with impressive versatility^12^,^13^,^14^ for performing fault-tolerant logic gates^15^,^16^,^17^. This is an important consideration as we search for schemes that realize fault-tolerant quantum computation with a low cost in quantum resources. In particular, a fault-tolerant quantum computer must be able to perform a non-Clifford operation, such as the *π*/8-gate, to realize universal quantum computation.

In general, performing Non-Clifford gates can present a considerable resource cost over the duration of a quantum computation. As such, the resource cost of realizing scalable quantum computation is sensitive to the method a fault-tolerant computational scheme uses to realize non-Clifford gates. To this end, the gauge color code^7^,^18^,^19^,^20^ has attracted significant recent interest because, notably, this three-dimensional quantum error-correcting code can achieve a universal gate set via gauge fixing^21^,^22^.

In contrast to the gauge-color code, surface code quantum computation, a leading approach towards low-resource quantum computation^8^,^9^,^23^, makes use of magic state distillation^24^ to perform *π*/8-gates. Magic state distillation can be achieved with 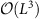 space-time resource cost^23^,^25^,^26^,^27^,^28^. Similarly, the gauge color code performs *π*/8-gates via gauge fixing in constant time^19^ and, as such, has an equivalent scaling in space-time resource cost as the surface code, as the gauge color code requires 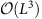 physical qubits. However, given that gauge fixing requires no additional offline quantum resources to perform a non-Clifford rotation, the gauge color code may reduce the quantum resources that are necessary for fault-tolerant quantum computation by a constant fraction.

It is also noteworthy that the gauge color code is local only in three dimensions and, as such, unlike the surface code, cannot be realized using a two-dimensional array of locally interacting qubits. Instead, the gauge color code may be an attractive model for non-local quantum-computational architectures such as networked schemes^29^,^30^,^31^,^32^,^33^.

Given the significant qualitative differences between the gauge color code and the surface code, it is interesting to perform a comparative analysis of these two proposals. In this study we investigate error correction with the gauge color code.

Dealing with errors that continually occur on physical qubits is particularly difficult in the realistic setting where syndrome measurements can fail and return false readings^9^. Attempting to correct errors using inaccurate syndrome information will introduce new physical errors to the code. However, given enough syndrome information, we can distinguish measurement errors from physical errors with enough confidence that the errors we introduce are few and can be identified at a later round of error correction^19^. In the case of the toric code^9^, we accumulate sufficient error data by performing multiple rounds of syndrome measurements. Surprisingly, the structure of the gauge color code enables the acquisition of fault-tolerant syndrome data using only one round of local measurements^19^. This capability is known as single-shot error correction.

Here we obtain a noise threshold for the gauge color code using a phenomenological noise model where both physical errors and measurement faults occur at rate *p*. We develop a single-shot decoder to identify the sustainable operating conditions of the code, that is, the noise rate below which information can be maintained arbitrarily well, even after many cycles of error correction. We estimate a sustainable error rate of *p*_sus_∼0.31% using an efficient clustering decoding scheme^34^,^35^,^36^,^37^,^38^,^39^ that runs in time 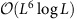
35, where the distance of the code is *d*=*L*+2 (ref. 7). Remarkably, the threshold we obtain falls within an order or magnitude of the optimal threshold for the toric code under the same error model, ∼2.9% (ref. 40). Furthermore, we also use our decoder to estimate how the logical failure rate of the gauge color code scales below the threshold error rate by fitting to a heuristic scaling hypothesis.

## Results

### The gauge color code

The gauge color code is a subsystem code^41^ specified by its gauge group, 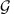. From the centre of the gauge group, 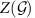, we obtain the stabilizer group for the code, 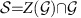 and its logical operators 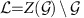. Elements of the stabilizer group, 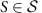, satisfy the property that 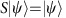 for all codewords of the code 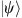.

**Figure 1:**
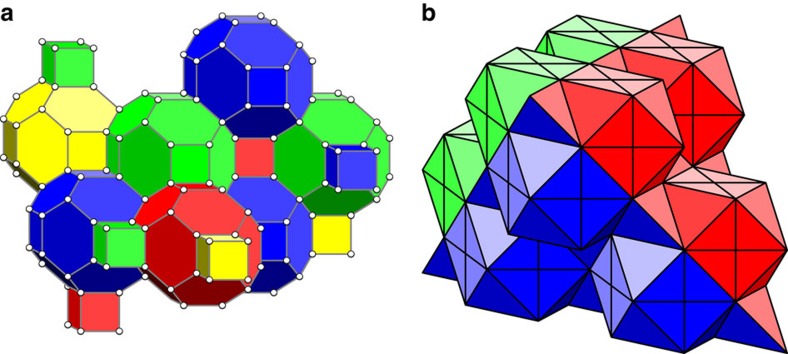
Representations of the lattice geometry of the gauge color code. (**a**) In the primal picture qubits sit on the vertices of a four-valent lattice. The three-dimensional cells of the lattice are four colorable, that is, every cell can be assigned one of four colors such that it touches no other cell of the same color. (**b**) The gauge color code of linear size *L*=5 drawn in the dual picture. Qubits lie on simplices of the lattice. The faces of the tetrahedra in the figure are given one of four colors such that no two faces of a given tetrahedron have the same color. The tetrahedra are stacked such that faces that touch always have the same color. For the gauge color code to encode a single logical qubit, the lattice must have four distinct, uniformly colored boundaries, as is shown in the figure. We give more details on the lattice construction in the dual picture in Supplementary Note 1 together with Supplementary Figs 1–6.

The code is defined on a three-dimensional four-valent lattice of linear size *L* with qubits on its vertices^4^,^42^. The lattice must also be four-colorable, that is, each cell of the lattice can be given a color, red, green, yellow or blue, denoted by elements of the set 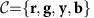, such that no two adjacent cells are of the same color. The lattice we consider is shown in Fig. 1a, where the cells are the solid colored objects in the figure.

The cells of the lattice define stabilizer generators for the code, whereas the faces of cells define its gauge generators. The gauge operators, otherwise known as face operators, are measured to infer the values of stabilizer operators. The face operators have weight four, six and eight, where the weight-eight face operators lie on the boundary of the lattice. More specifically, for each cell *c* there are two stabilizer generators, 
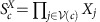
 and 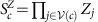, where 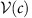 are the qubits on the boundary vertices of cell *c*, and *X*_*j*_ and *Z*_*j*_ are Pauli-X and Pauli-Z operators acting on vertex *j*. For each face *f*, there are two face operators, 
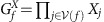
 and 
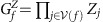
, where 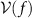 are the vertices on the boundary of face *f*. We will call the outcome of a face operator measurement a face outcome.

Given suitable boundary conditions^7^,^42^, the code encodes one qubit, whose logical operators are 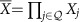 and 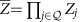, where 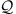 is the set of physical qubits of the code. A lattice with correct boundaries is conveniently represented on a dual lattice of tetrahedra using the convention given in ref. 7. The lattice we consider is illustrated in Fig. 1b, where we discuss its construction in detail in the Supplementary Notes 1 and 2, together with Supplementary Figs 1–7. Importantly, we require that the lattice has four boundaries, distinguished by colors from set 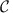.

### Single-shot error correction

A quantum error-correcting code is designed to identify and correct errors. Owing to the symmetry of the gauge group, it suffices here to consider only bit-flip, that is, Pauli-X errors. We consider a phenomenological error model consisting of physical errors and measurement errors. A physical error is a Pauli-X error on a qubit, whereas a measurement error returns the opposite outcome of the correct reading. Errors will be identically and independently generated with the same probability *p*.

In a stabilizer code, errors are identified by stabilizer measurements that return eigenvalue −1, which we call stabilizer defects. We use the stabilizer syndrome, a list of the positions of stabilizer defects, to predict the incident error. In the gauge color code we do not measure stabilizer operators directly, but instead infer their values by measuring face operators, which is possible due to the fact that 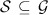.

In addition to using face outcomes to infer stabilizer eigenvalues, we can also exploit the local constraints in 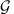 of the gauge color code to detect and account for measurement errors. Remarkably, measurement errors can be detected reliably by measuring each face operator only once, so-called single-shot error correction^19^.

**Figure 2:**
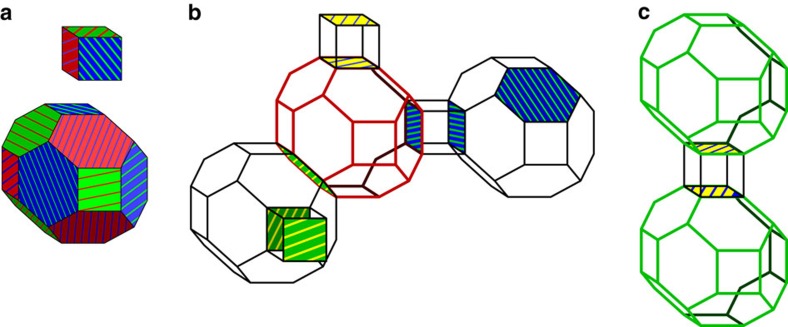
The gauge syndrome drawn on the primal lattice. (**a**) The faces of the cells of the color code are three colorable. (**b**) An example of a set of face measurement outcomes where measurements are reliable. Face operators that return value −1 are colored, otherwise they are left transparent. The stabilizer at the cell with thick red edges contains a stabilizer defect. This cell has one face operator of each color returning a −1 outcome. All other cells have an even parity of −1 face outcomes over their colored subsets, indicating that these cells contain no stabilizer defect. (**c**) A gauge syndrome where colored subsets of face measurements about the green cells do not agree, thus indicating measurement errors.

The local constraints stem from the structure of the code. We first observe that the faces of a cell are necessarily three color able, as shown in Fig. 2a. It follows from the three colorability of the faces that the product of the gauge operators 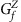 of any of the differently colored subsets of faces of cell *c* recover the stabilizer operator 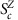. By measuring all the faces of the lattice, we redundantly recover each stabilizer eigenvalue three times where the three outcomes of a given cell are constrained to agree. In Fig. 2b we show an example of a gauge measurement configuration where the outcomes are reliable. Following this observation, we can use violations of the local constraints about a cell to indicate the positions of measurement errors. In Fig. 2c we show a syndrome where the product of the face outcomes of different subsets of of two cells, colored with thick green edges, do not agree.

Fault-tolerant decoding with the gauge color code proceeds in two stages. The first stage, syndrome estimation, uses face outcomes that may be unreliable to estimate the locations of stabilizer defects. The second stage, stabilizer decoding, takes the estimated stabilizer defect locations and predicts a correction operator to reverse physical errors. The latter is discussed extensively in refs 4, 42 and can be dealt with using standard decoding methods^35^,^43^,^44^. To perform stabilizer decoding we adapt a clustering decoder^34^,^35^,^36^,^37^,^38^,^39^ where clusters grow linearly.

We concentrate now on syndrome estimation. Syndrome estimation uses a gauge syndrome, a list of gauge defects, to estimate a stabilizer sydrome. A lattice cell can contain as many as three gauge defects. Gauge defects are distinguished by a color pair **uv**, with 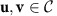, such that **u**≠**v** and **uv**=**vu**. The color pair of a gauge defect relates to the coloring of the lattice faces. A face is given the color pair opposite to the colors of its adjacent cells, that is, the face shared by two cells with colors **r** and **g** is colored **yb**. A cell *c* contains a **uv** gauge defect if the product of all the **uv** face outcomes bounding *c* is −1. Following this definition, a stabilizer defect is equivalent to three distinct gauge defects in a common cell.

Studying the gauge syndrome enables the identification of measurement errors. We consider face *f*, colored **uv**, that is adjacent to cell *c*. In the noiseless measurement case, where *c* contains no stabilizer defect, by definition, cell *c* should contain no gauge defects. However, if face *f* returns an incorrect outcome, we identify a **uv** gauge defect at *c*. Conversely, if cell *c* contains a stabilizer defect in the ideal case and *f* returns an incorrect outcome, no **uv** gauge defect will appear in *c*. With these examples we see that cells that contain either one or two gauge defects indicate incorrect face outcomes.

An incorrect face outcome affects gauge defects in both of its adjacent cells. In general, incorrect face outcomes of color **uv** form error strings on the dual lattice, whose end points are **uv** gauge defects, where individual incorrect face outcomes are segments of the string. Error strings of incorrect **uv** face outcomes changes the parity of **uv** gauge defects at both of its terminal cells.

We require an algorithm that can use gauge syndrome data to predict a probable measurement error configuration and thus estimate the stabilizer syndrome. We adapt the clustering decoder^34^,^35^,^36^,^37^,^38^ for this purpose. The decoder combines nearby defects into clusters that can be contained within a small box. Clusters increase linearly in size to contain other nearby defects until they contain a set of gauge defects that can be caused by a measurement error contained within the box. Once clustering is completed, a correction supported inside the boxes is returned.

**Figure 3:**
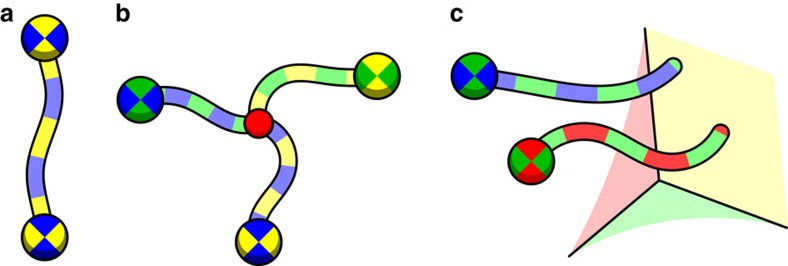
The gauge defects shown in the dual picture. (**a**) A pair of **yb** gauge defects, shown by the vertices, can be caused by a string of incorrect **yb** face outcomes on the dual lattice. The displayed gauge syndrome is equivalent to that shown in Fig. 2c where the measurement errors have occurred on the faces that returned −1 outcomes. (**b**) Three gauge defects, colored **gy**, **gb** and **yb**, can be caused by an error string that branches at a red cell. The branching point indicates a stabilizer defect at a red cell. (**c**) Strings of incorrect face outcomes of colors **rg** and **gb** terminate at a yellow boundary.

We briefly elaborate on correctable configurations of gauge defects. Pairs of **uv** gauge defects are caused by strings of incorrect **uv** face outcomes and therefore form correctable configurations, as shown in Fig. 3a. As an example, the gauge syndrome in Fig. 3a depicts the gauge defects and measurement error string shown in Fig. 2c where the measurement errors have occurred on the faces that returned −1 measurement outcomes. Triplets of gauge defects, colored **uv**, **vw** and **uw**, also form correctable configurations. Error strings that cause triplets of correctable gauge defects branch at a cell, and thus indicate a stabilizer defect, shown in Fig. 3b. The stabilizer defect where the error string branches lies at a cell colored **x**≠**u**, **v**, **w**. Gauge defects can also arise due to incorrect face outcomes on the lattice boundary. Specifically, the boundary colored **w** contains faces of color **uv**, where **u**, **v**≠**w**. With this coloring we can find correctable configurations of single **uv** gauge defects, together with a boundary of color **w** (see Fig. 3c). In general, a cluster can contain many correctable pairs and triplets of gauge defects.

As we have mentioned, correctable clusters of gauge defects can give rise to stabilizer defects. It is important to note that the error-correction procedure is sensitive to the positions of stabilizer defects within a correctable cluster, as discrepancies in their positions later affect the performance of the stabilizer decoding algorithm. As such, we must place stabilizer defects carefully. For cases where a correctable cluster of gauge defects returns stabilizer defects, we assign their positions such that they lie at the mean position of all the gauge defects within the correctable cluster, at the nearest cell of the appropriate color. Once syndrome estimation is complete, the predicted stabilizer syndrome is passed to the stabilizer decoder and a correction operator is evaluated.

We remark that gauge defects can be incorrectly analysed during syndrome estimation. In which case, measurement errors sometimes masquerade as stabilizer defects and sometimes stabilizer defects can be misplaced. We will then attempt to decode the incorrect stabilizer syndrome and mistakenly introduce errors to the code. In general, any error-correction scheme that takes noisy measurement data will introduce residual physical errors to a code. These errors can be corrected in future cycles of error-correction, provided the remaining noise is of a form that a decoder can correct. In general, however, one must worry that large correlated errors can be introduced that adversarially corrupt encoded information^45^,^46^,^47^,^48^,^49^,^50^. Such errors may occur in the gauge color code if, for instance, we mistakenly predict two stabilizer defects of the same color that are separated by a large distance. We give an example of a mechanism that might cause a correlated error during syndrome estimation with the gauge color code in the Supplementary Note 3, together with Supplementary Fig. 8.

A special property of the gauge color code is that measurement errors, followed by syndrome estimation, will only introduce false defects in locally correctable configurations. Therefore, residual errors remain local to the measurement error. Moreover, the code is such that the probability of obtaining configurations of face outcomes that correspond to faux stabilizer defects decays exponentially with the separation of their cells. This is because the number of measurement errors that must occur to produce a pair of false stabilizer defects is extensive with their cell separation. To this end, the errors introduced from incorrect measurements are local to the measurement error and typically small. This property, coined ‘confinement' in ref. 19, is essential for fault-tolerant error correction. Most known codes achieve confinement by performing syndrome measurements many times. We give numerical evidence showing that our error-correction protocol confines errors in the following subsection.

### The simulation

We simulate fault-tolerant error correction with encoded states 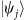 of linear size *L* where *j* indicates the number of error-correction cycles that have been performed and where 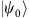 is a codeword. We seek to find a correction operator *C* such that 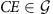 where *E* is the noise incident to 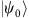 after *N* error-correction cycles.

To maintain the encoded information over long durations, we repeatedly apply error-correction cycles to keep the physical noise sufficiently benign. After a short period, the state 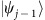 will accumulate physical noise *E*_*j*_(*p*) with error rate *p*. To correct the noise, we first estimate a stabilizer syndrome, **s**_*j*_, using gauge syndrome data with the syndrome estimation algorithm *M*_*q*_, where measurement outcomes are incorrect with probability *q*=*p*. Specifically, we have that 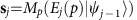. We then use the stabilizer decoding algorithm *D* to predict a suitable correction operator *C*_*j*_=*D*(**s**_*j*_), such that we obtain


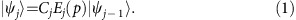


It is important to note that 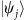 is not necessarily in the code subspace. For *q*>0, stabilizer syndromes will in general be incorrectly estimated and thus the correction operator *C*_*j*_ introduces some new errors to the code.

We require that after *N* error-correction cycles we can estimate error *E* of state 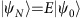 to perform a logical measurement. To perform the 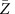 logical measurement^19^,^51^, we measure each individual qubit of the code in the Pauli-Z basis. This destructive transversal measurement gives us the eigenvalues of stabilizers 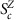 to diagnose *E* and to thus recover the eigenvalue of 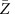.

During readout, measurement errors and physical errors have an equivalent effect; both appear as bit flips. To simulate errors that occur during the readout process, we apply the noise operator *E*(*p*) to the encoded state before decoding. We therefore calculate logical failure rates


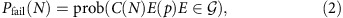


where 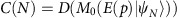. We evaluate *P*_fail_(*N*) values using Monte Carlo simulations.

To analyse the performance of the proposed decoding scheme, we first look to find the sustainable error rate of the code, *p*_sus_, below which we can maintain quantum information for an arbitrary number of correction cycles. The discovery of such a point suggests that the error correlations caused by our correction protocol do not extend beyond a constant, finite and decodable length, thus showing that we can preserve information indefinitely with arbitrarily high fidelity in the *p*<*p*_sus_ regime.

**Figure 4:**
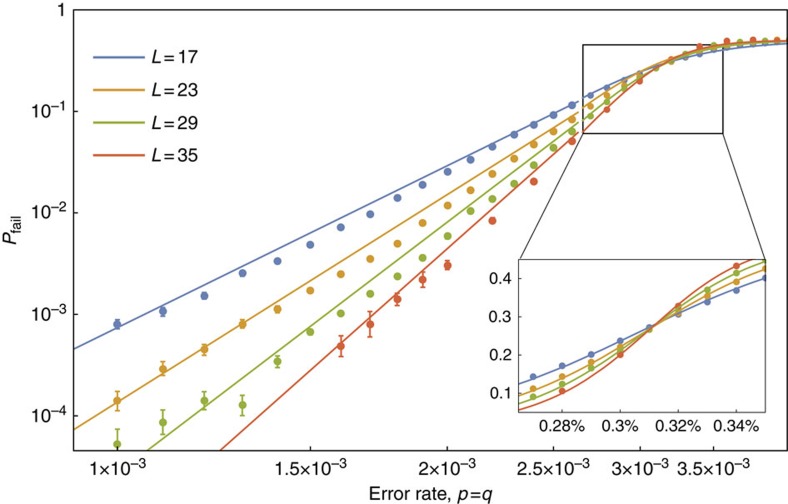
Gauge color code logical failure rates plotted as a function of physical error rate. We show logical error rates, *P*_fail_, as a function of physical error rate, *p*, for system sizes *L*=17, 23, 29 and 35 shown in blue, yellow, green and red, respectively, as is marked in the legend, where we collect data after *N*=8 rounds of error correction during which measurements are performed unreliably. The error bars show the standard error of the mean given by the expression 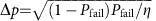 where η is the number of Monte Carlo samples we collect. The data used to determine the threshold error rate is shown in the inset, where we determine the threshold using the fitting described in the Methods. The fitting is also plotted in the inset. In the main Figure the solid lines show the fitted expression, equation (4), to demonstrate the agreement of our scaling hypothesis with numerically evaluated logical error rates where *p*<*p*_th_. We remark that the fitting is made using data for all values of *N*, and not only the data shown in this plot, as we explain in the Methods.

We define *p*_sus_ as the threshold error rate, *p*_th_, at the *N*→∞ limit, where the threshold is the error rate below which we can decrease *P*_fail_ arbitrarily by increasing *L*^10^. In the inset of Fig. 4 we show the near-threshold data we use to evaluate a threshold, where we show the data for *N*=8 as an example. We give the details of the fitting model we use to evaluate thresholds in the Methods.

**Figure 5:**
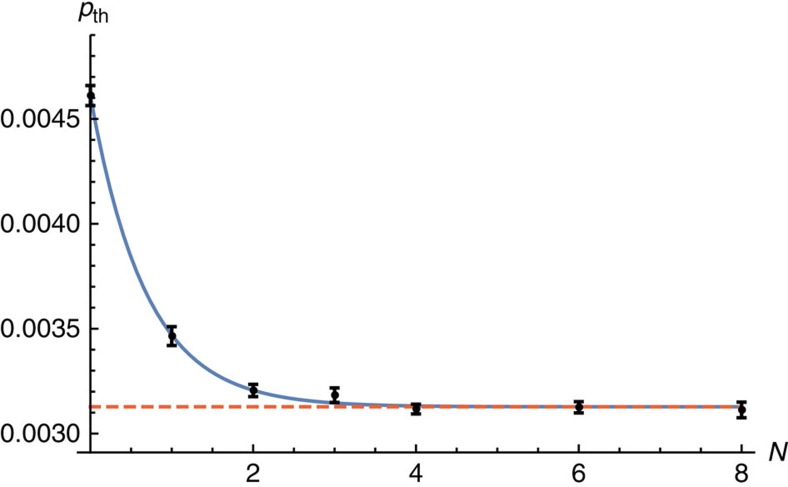
Gauge color code threshold error rates plotted as a function of the number of error-correction cycles. Threshold error rates, *p*_th_, are calculated with system sizes *L*=23, 29, 35 after *N* error-correction cycles using *η*∼10^4^ Monte Carlo samples. Error bars show the standard error of the mean which are determined using the NonLinearModelFit function in Mathematica. The solid blue line shows the fitting given in equation (3). The dashed red line marks *p*_sus_∼0.31%, the sustainable noise rate of the code, the limiting value of *p*_th_ from to the fitting as *N*→∞.

We next study the evaluated threshold values as a function of *N*. We show these data in the plot given in Fig. 5. The data show that *p*_th_ converges to *p*_sus_∼0.31% where we fit for values *N*≤8. We obtain this value with a fitting that converges to *p*_sus_, namely


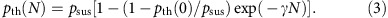


We find *p*_th_(0)∼0.46% and *γ*∼1.47. The convergent trend provides evidence that we achieve steady-state confinement in the high-*N* limit, as is required of a practical error-correction scheme.

To verify further the threshold error rates we have determined, we next check that the logical failure rate decays as a function of system size in the regime where *p*<*p*_th_. In the large *N* limit, we fit our data to the following hypothesis


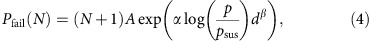


where *A*, *α* and *β* are positive constants to be determined and *d* is the distance of the code. We evaluate the variables in equation (4) as *A*∼0.033, *α*∼0.516 and *β*∼0.822 using ∼5,000 CPU hours with data for *N*≤10. Details on the fitting calculation are given in Methods. We plot the fitted scaling hypothesis on Fig. 4 for the case of *N*=8, to show the agreement of equation (4) with the available data.

## Discussion

To summarize, using only a simple decoding scheme, we have obtained threshold values that lie within an order of magnitude of the optimal threshold for the toric code under the phenomenological noise model. Moreover, we can expect that higher thresholds are achievable using more sophisticated decoding strategies^36^,^37^,^43^,^44^,^52^,^53^,^54^,^55^,^56^,^57^,^58^. It may be possible to achieve a sufficiently high sustainable noise rate to become of practical interest, thus meriting comparison with the intensively studied surface code^59^,^60^. To this end, further investigation is required to learn its experimental viability.

To continue such a comparative analysis, one should study the code using realistic noise models^25^ that respect the underlying code hardware. We expect that the threshold will suffer relative to the surface code when compared using a circuit-based noise model where high-weight gauge measurements are more error prone^61^. Fortunately, gauge color code lattices are known where face operators have weight no greater than 6 (ref. 7). Although this is not as favourable as the weight-four stabilizer measurements of the surface code, given the ability to perform single-shot error correction, and *π*/8-gates through gauge fixing, we argue that the gauge color code is deserved of further comparison.

To the best of our knowledge, we have obtained the first threshold using single-shot error correction. Fundamentally, our favourable threshold is achieved using redundant syndrome data to identify measurement errors. It is interesting to ask whether we can make use of a more intelligent collection of measurement data to improve thresholds further. Discovering single-shot error-correction protocols with simpler codes might help to address such questions.

## Methods

### Threshold calculations

The threshold error rate, *p*_th_, is the physical error rate below which the logical failure rate of the code can be arbitrarily suppressed by increasing the code distance. We identify thresholds by plotting the logical failure rate as a function of physical error rate *p* for several different system sizes and identify the value *p*=*p*_th_ such that *P*_fail_ is invariant under changes in system size. In the main text, we show the data used for a specific threshold calculation in Fig. 4 where we use the *N*=8 data as an example, and in Fig. 5 we evaluate threshold error rates for the gauge color code as a function of the number of error correction cycles, *N*.

We evaluate threshold error rates by performing *η*∼10^4^ Monte Carlo simulations for each value of *p* close to the crossing point using codes of system sizes *L*=23, 29 and 35, except for the case that *N*=0 where we evaluate the logical failure rate with system sizes *L*=31, 39 and 47. Simulating larger system sizes where *N*=0 is possible as in this case we read out information immediately after encoding it, such that *E*=1 as shown in equation (2). We therefore need not perform syndrome estimation in the *N*=0 simulation. The threshold error rate at *N*=0 is thus the threshold error rate of the clustering decoder for the gauge color code where measurements are performed perfectly, that is, *q*=0.

We identify the crossing point by fitting our data to the following formula


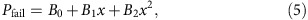


where *x*=(*p*−*p*_th_)*L*^1/*μ*^ and *B*_*j*_, *p*_th_ and *μ* are constants to be determined. We show an example of this fitting in the inset of Fig. 4.

At the threshold error rate the code produces logical failures at a rate between 0.075 and 0.27 depending on *N*. We expect such behaviour as the number of logical failures will increase with repeated use of a decoder. We therefore obtain between ∼750 and ∼2,700 logical failures per data point close to the threshold error rate.

### Overhead analysis

Here we summarize the resource-scaling analysis we give in the *p*<*p*_th_ regime. We suppose the logical failure rate in this regime scales such as


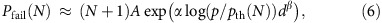


where *A*, *α* and *β* are constants to be determined, *d* is the distance of the code, *p* is the error rate and *N* is the number of uses of the decoder we make before readout. The value of *p*_th_(*N*) is determined by the method given in the previous subsection. This equation is derived by assuming that a single use of the decoder will fail with probability 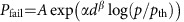 in the low-*p* regime. We then calculate to first order the probability that the decoder will fail a single time in *N*+1 uses to give equation (6), where we include an additional use of the decoder to account for a possible logical failure during readout.

We manipulate equation (6) to show a method to evaluate α and β. We first take the logarithm of both sides of equation (6) to find the linear expression


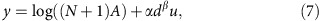


where we write *y*=log *P*_fail_ and *u*=log (*p*/*p*_th_). We then take the gradient, *g*(*d*)=*dy*/*du*, from equation (7) to find


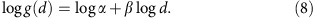


**Figure 6:**
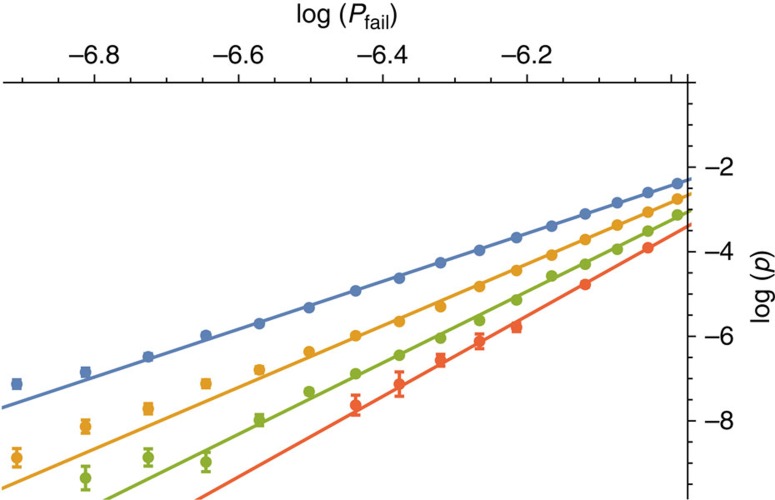
Logical failure rates for the gauge color code plotted as a function of physical error rate. Plot showing log *P*_fail_ as a function of *logp* for the case that *N*=8 where we have plotted system sizes *L*=17, 23, 29 and 35 shown in blue, yellow, green and red, respectively. The error bars show the s.e.m. given by the expression 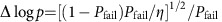 where *η* is the number of Monte Carlo samples we collected. The logarithm of the gradients found for the linear fittings are plotted as a function of log *d* in Fig. 7.

**Figure 7:**
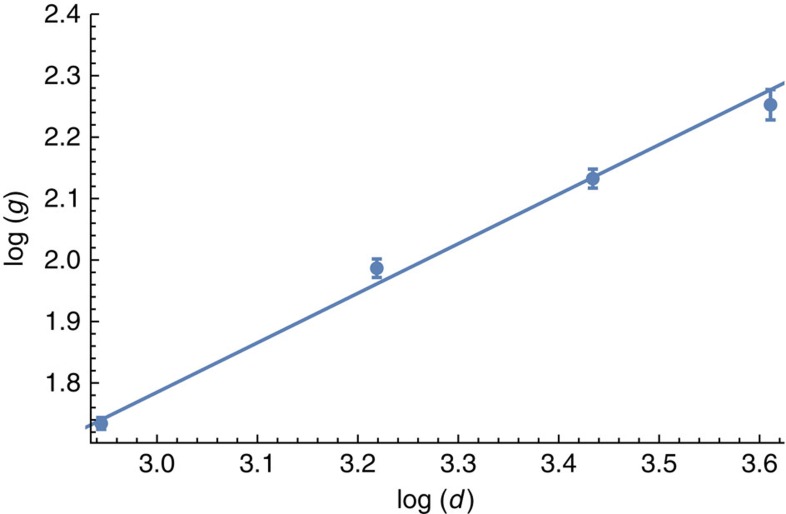
Linear fitting used to determine *α* and *β*. Plot shows the available data fitted to the trend anticipated in equation (8). The figure shows the logarithm of the gradients found in Fig. 6, log *g*(*d*), plotted as a function of the logarithm of the code distance, log *d*, for the case where *N*=8. Error bars show the s.e.m., evaluated using the LinearModelFit function in Mathematica. For this case we obtain a fitting log *g*(*d*)≈0.81 log *d*−0.63, as is shown in the plot.

In Fig. 6 we plot log *P*_fail_ as a function of log *p*. For fixed system sizes we observe a linear fitting that equation (7) predicts. We take the gradient of each of these fittings to estimate *g*(*d*), as given in equation (8). Next, to find *α* and *β*, we plot log *g*(*d*) as a function of log *d* where the gradients are taken from the fittings shown in Fig. 6. This data is shown in Fig. 7. We can now determine *α* and *β* using, respectively, the *d*=1 intersection and the gradient of a linear fit shown in Fig. 7.

The logical failure rates we use to find *α* and *β* are found using between 10^5^ and 10^6^ Monte Carlo samples for each value of *p* where we only take values of *P*<0.8 × *p*_th_ for each *N*. We discard data points where we observe fewer than ten failures for a given *p*. The data were collected over 5,000 CPU hours.

**Figure 8:**
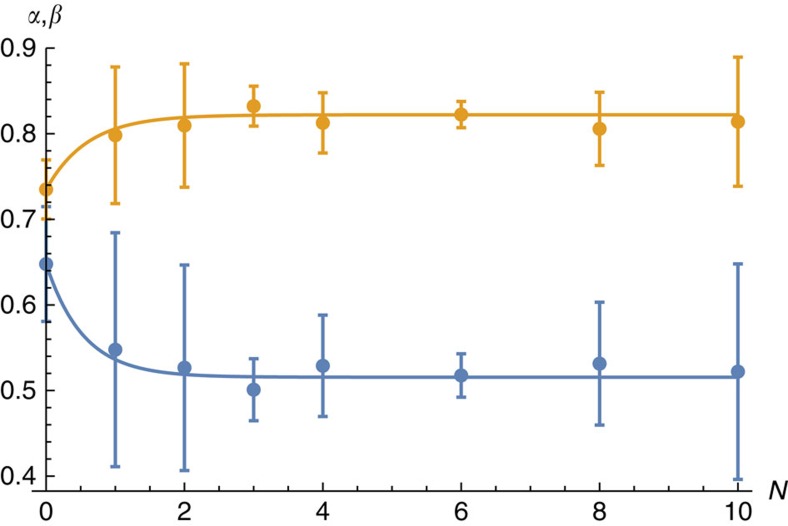
Variation in constant values *α* and *β* plotted against the number of error-correction cycles. Unitless values, *α* and *β*, plotted as a function of error-correction cycles, *N*, shown in blue and yellow, respectively. The error bars show the standard error of the mean, which are found using the NonLinearModelFit function included in Mathematica. The *α* and *β* data points are fitted to equations (9) and (10), respectively. The fitted functions are shown in the plot.

We plot the values we find for *α* and *β* as a function of *N* in Fig. 8. Importantly, we observe convergence in the large *N* limit. We see this using the fitting functions


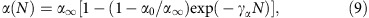


and


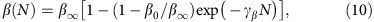


to find the *N*→∞ behaviour of our protocol, where *α*_0_, *β*_0_, *α*_∞_, *β*_∞_, *γ*_*α*_ and *γ*_*β*_ are constants to be determined, such that


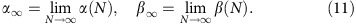


We fit these functions to our data to find


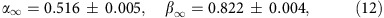


together with the following values *α*_0_=0.65±0.02, *γ*_*α*_=1.9±1.5, *β*_0_=0.73±0.01 and *γ*_*β*_=1.7±1.4. The fittings are shown in Fig. 8.

**Figure 9:**
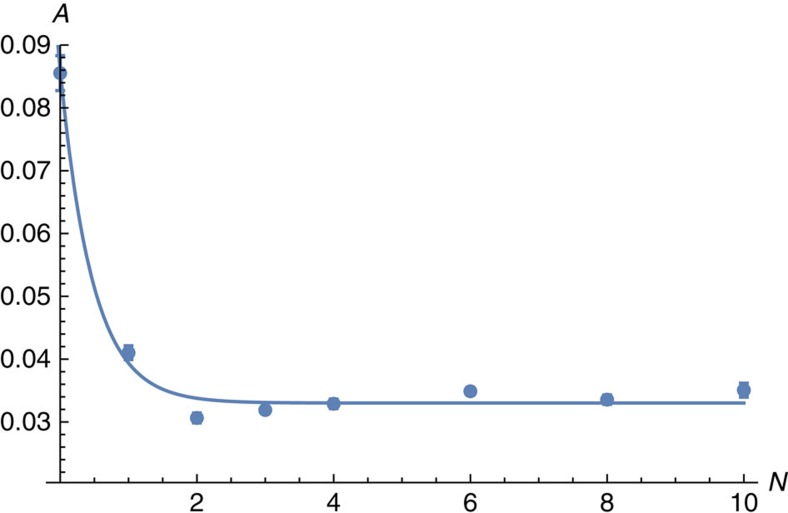
Variation in the constant A plotted as a function of the number of applied error-correction cycles. Figure shows unitless values of *A* numerically determined as a function of the number of error-correction cycles we perform, *N*. The error bars show the standard error of the mean which is calculated using Mathematica. We fit the data to equation (13) as we show in the figure.

Finally, given that we have evaluated *α*(*N*) and *β*(*N*) for different values of *N*, we use these values together with equation (6) to determine *A* as a function of *N*. We show the data in Fig. 9. We fit the values of *A*(*N*) to the following expression


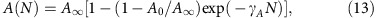


to find values *A*_∞_=0.033±0.001, *A*_0_=0.09±0.01 and *γ*_*A*_=2.1±0.5, thus giving all of the variables, *A*_∞_, *α*_∞_ and *β*_∞_, we require to estimate the *N*→∞ behaviour of the decoding scheme in the below threshold regime.

### Data availability

The code and data used in this study are available upon request to the corresponding author.

## Additional information

**How to cite this article:** Brown, B. J. *et al*. Fault-tolerant error correction with the gauge color code. *Nat. Commun.* 7:12302 doi: 10.1038/ncomms12302 (2016).

## Supplementary Material

Supplementary Information Supplementary Figures 1-8 and Supplementary Notes 1-3.

## References

[b1] Shor P. W. Scheme for reducing decohernece in quantum computer memory. Phys. Rev. A 52, 2493 (1995). 10.1103/physreva.52.r2493 9912632

[b2] Steane A. Error-correcting codes in quantum theory. Phys. Rev. Lett. 77, 793–796 (1996). 10062908 10.1103/PhysRevLett.77.793

[b3] Bombin H. & Martin-Delagado M. A. ‘Topological quantum distillation,'. Phys. Rev. Lett. 97, 180501 (2006). 17155532 10.1103/PhysRevLett.97.180501

[b4] Bombin H. & Martin-Delgado M. A. Exact topological quantum order in *d*=3 and beyond: Branyons and brane-net condensates. Phys. Rev. B 75, 075103 (2007).

[b5] Bombin H. Topological subsystem codes. Phys. Rev. A 81, 032301 (2010).

[b6] Bombin H., Chhajlany R. W., Horodecki M. & Martin-Delagado M. A. Self-correcting quantum computers. New J. Phys. 15, 055023 (2013).

[b7] Bombín H. Gauge color codes: optimal transversal gates and gauge fixing in topological stabilizer codes. New J. Phys. 17, 083002 (2015).

[b8] Kitaev A. Y. Fault-tolerant quantum computation by anyons. Ann. Phys. 303, 2–30 (2003).

[b9] Dennis E., Kitaev A., Landahl A. & Preskill J. Topological quantum memory. J. Math. Phys. 43, 4452–4505 (2002).

[b10] Terhal B. M. Quantum error correcton for quantum memories. Rev. Mod. Phys. 87, 307 (2015).

[b11] Lidar D. A., Brun T. A. (eds) in Quantum Error Correction Cambridge Univ. Press (2013).

[b12] Eastin B. & Knill E. Restrictions on transveral encoded quantum gate sets. Phys. Rev. Lett. 102, 110502 (2009). 19392181 10.1103/PhysRevLett.102.110502

[b13] Bravyi S. & König R. Classification of topologically protected gates for local stabilizer codes. Phys. Rev. Lett. 110, 170503 (2013). 23679695 10.1103/PhysRevLett.110.170503

[b14] Kubica A., Yoshida B. & Pastawski F. Unfolding the color code. New J. Phys. 17, 083026 (2015).

[b15] Bombin H. Clifford gates by code deformation. New J. Phys. 13, 043005 (2011).

[b16] Fowler A. G. Two-dimensional color-code quantum computation. Phys. Rev. A 83, 042310 (2011).

[b17] Landahl A. J. & Ryan-Anderson C. Quantum computing by color-code lattice surgery. Preprint at http://arXiv.org/abs/1407.5103 (2014).

[b18] Kubica A. & Beverland M. E. Universal transversal gates with color codes - a simplified approach. Phys. Rev. A 91, 032330 (2015).

[b19] Bombín H. Single-shot fault-tolerant quantum error correction. Phys. Rev. X 5, 031043 (2015).

[b20] Bombín H. Dimensional jump in quantum error correction. New J. Phys. 18, 043038 (2016).

[b21] Paetznick A. & Reichardt B. W. Universal fault-tolerant quantum computation with only transversal gates and error correction. Phys. Rev. Lett. 111, 090505 (2013). 24033013 10.1103/PhysRevLett.111.090505

[b22] Anderson J. T., Duclos-Cianci G. & Poulin D. Fault-tolerant conversion between the Steane and Reed-Muller quantum codes. Phys. Rev. Lett. 113, 080501 (2014). 25192082 10.1103/PhysRevLett.113.080501

[b23] Raussendorf R., Harrington J. & Goyal K. Topological fault-tolerance in cluster state quantum computation. New J. Phys. 9, 199 (2007).

[b24] Bravyi S. & Kitaev A. Universal quantum computation with ideal Clifford gates and noisy ancillas. Phys. Rev. A 71, 022316 (2005).

[b25] Fowler A. G., Mariantoni M., Martinis J. M. & Cleland A. N. Surface codes: towards practical large-scale quantum computation. Phys. Rev. A 86, 032324 (2012).

[b26] Bravyi S. & Haah J. Magic-state distillation with low overhead. Phys. Rev. A 86, 052329 (2012).

[b27] Meier A. M., Eastin B. & Knill E. Magic-state distillation with the four-qubit code. Quant. Inf. Comp. 13, 0195–0209 (2013).

[b28] Li Y. A magic state's fidelity can be superior to the operations that created it. New J. Phys. 17, 023037 (2015).

[b29] Barrett S. D. & Kok P. Efficient high-fidelity quantum computation using matter qubits and linear optics. Phys. Rev. A 71, 060310 (R) (2005).

[b30] Fujii K., Yamamoto T., Koashi M. & Imoto N. A distributed architecture for scalable quantum computation with realistically noisy devices. Preprint at http://arXiv.org/abs/1202.6588 (2012).

[b31] Nickerson N. H., Li Y. & Benjamin S. C. Topological quantum computing with a very noisy network and local error rates approaching one percent. Nat. Commun. 4, 1756 (2013). 23612297 10.1038/ncomms2773 PMC3644110

[b32] Monroe C. . Large-scale modular quantum-computer architectures with atomic memory and photonic interconnects. Phys. Rev. A 89, 022317 (2014).

[b33] Nickerson N. H., Fitzsimons J. F. & Benjamin S. C. Freely scalable quantum technologies using cells of 5-to-50 qubits with very lossy and noisy photonic links. Phys. Rev. X 4, 041041 (2014).

[b34] Harrington J. W. Analysis of Quantum Error-Correcting Codes: Sympletic Lattice Codes and Toric Codes PhD Thesis, (California Institute of Technology (2004).

[b35] Bravyi S. & Haah J. Quantum self-correction in the 3D cubic code model. Phys. Rev. Lett. 111, 200501 (2013). 24289671 10.1103/PhysRevLett.111.200501

[b36] Anwar H., Brown B. J., Campbell E. T. & Browne D. E. Fast decoders for qudit topological codes. New J. Phys. 16, 063038 (2014).

[b37] Hutter A., Loss D. & Wootton J. R. Improved HDRG decoders for qudit and non-abelian quantum error correction. New J. Phys. 17, 035017 (2015).

[b38] Watson F. H. E., Anwar H. & Browne D. E. A fast fault-tolerant decoder for qubit and qudit surface codes. Phys. Rev. A 92, 032309 (2015).

[b39] Wootton J. R. & Hutter A. Active error correction for Abelian and non-Abelian anyons. Phys. Rev. A 93, 022318 (2016).

[b40] Wang C., Harrington J. & Preskill J. Confniement-Higgs transition in a disordered gauge theory and the accuracy threshold for quantum memory. Ann. Phys. 303, 31–58 (2003).

[b41] Poulin D. Stabilizer formalism for operator quantum error correction. Phys. Rev. Lett. 95, 230504 (2005). 16384287 10.1103/PhysRevLett.95.230504

[b42] Bombin H. & Martin-Delgado M. A. Topological computation without braiding. Phys. Rev. Lett. 98, 160502 (2007). 17501406 10.1103/PhysRevLett.98.160502

[b43] Duclos-Cianci G. & Poulin D. Fast decoders for topological quantum codes. Phys. Rev. Lett. 104, 050504 (2010). 20366756 10.1103/PhysRevLett.104.050504

[b44] Wootton J. R. & Loss D. High threshold error correction for the surface code. Phys. Rev. Lett. 109, 160503 (2012). 23215062 10.1103/PhysRevLett.109.160503

[b45] Ahronov D., Kitaev A. & Preskill J. Fault-tolerant quantum computation with long-range correlated noise. Phys. Rev. Lett. 96, 050504 (2006). 16486913 10.1103/PhysRevLett.96.050504

[b46] Ng H. K. & Preskill J. Fault-tolerant quantum computation versus Gaussian noise. Phys. Rev. A 79, 032318 (2009).

[b47] Preskill J. Sufficient condition on noise correlations for scalable quantum computing. Quant. Inf. Comp. 13, 0181–0194 (2013).

[b48] Jouzdani P, Novais E., Tupitsyn I. S. & Mucciolo E. R. Fidelity threshold of the surface code beyond single-qubit error models. Phys. Rev. A 90, 042315 (2014).

[b49] Fowler A. G. & Martinis J. M. Quantifying the effects of local many-qubit errors and nonlocal two-qubit errors on the surface code. Phys. Rev. A 89, 032316 (2014).

[b50] Hutter A. & Loss D. Breakdown of surface-code error correction due to coupling to a bosonic bath. Phys. Rev. A 89, 042334 (2014).

[b51] Alicki R., Horodecki M., Horodecki P. & Horodecki R. On thermal stability of topological qubit in Kitaev's 4D model. Open Syst. Inf. Dyn. 17, 1–20 (2010).

[b52] Wang D. S., Fowler A. G., Hill C. D. & Hollenberg L. C. L. Graphical algorithms and threshold error rates for the 2D color code. Quant. Inf. Comp. 10, 0780–0802 (2010).

[b53] Bombin H., Duclos-Cianci G. & Poulin D. Universal topological phase of two-dimensional stabilizer codes. New J. Phys. 14, 073048 (2012).

[b54] Sarvepalli P. & Raussendorf R. Efficient decoding of topological color codes. Phys. Rev. A 85, 022317 (2012).

[b55] Hutter A., Wootton J. R. & Loss D. An efficient markov chain monte carlo algorithm for the surface code. Phys. Rev. A 89, 022326 (2014).

[b56] Delfosse N. Decoding color codes by projection onto surface codes. Phys. Rev. A 89, 012317 (2014).

[b57] Herold M., Campbell E. T., Eisert J. & Kastoryano M. J. Cellular-automation decoders for topological quantum memories. NPJ Quant. Inf. 1, 15010 (2015).

[b58] Stephens A. M. Efficient fault-tolerant decoding of topological color codes. Preprint at http://arXiv.org/abs/1402.3037 (2014).

[b59] Fowler A. G. Optimal complexity correction of correlated errors in the surface code. Preprint at http://arXiv.org/abs/1310.0863 (2013).

[b60] Fowler A. G. Minimum weight perfect matching of fault-tolerant topological quantum error-correction in average *O*(1) parallel time. Quant. Inf. Comp. 15, 0145–0158 (2015).

[b61] Landahl A. J., Anderson J. T. & Rice P. R. Fault-tolerant quantum computing with color codes. Preprint at http://arXiv.org/abs/1108.5738 (2011).

