# Supplementary material for: Fault-tolerant error correction with the gauge color code
Source: Nat Commun. 2016 Jul 29;7:12302. doi: 10.1038/ncomms12302 (PMC4974574; doi:10.1038/ncomms12302)
Supplement: Supplementary Information — Supplementary Figures 1-8 and Supplementary Notes 1-3. [file ncomms12302-s1.pdf]

SUPPLEMENTARY FIGURES

Supplementary Figure 1

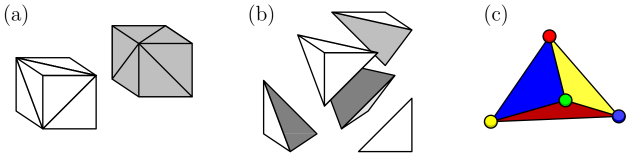

(a) An odd and an even unit cube that each consist of five tetrahedra. The odd unit cube differs from the even unit cube by a $\pm\pi/2$ rotation about any of the canonical axes. (b) An exploded even unit cell that reveals the internal structure of a unit cube. (c) A fundamental tetrahedron of the lattice whose vertices are correctly four colored. We color each face of every tetrahedron with the color that is not the color of any of its three vertices. The face that is not visible is colored green.

Supplementary Figure 2

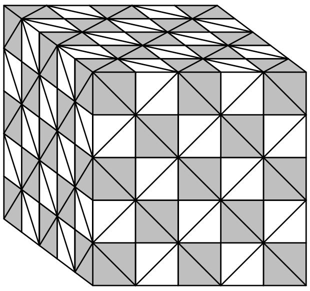

A cube of linear size $L = 5$ formed by stacking odd and even unit cubes. The odd and even unit cubes, shown in gray and white, are stacked such that no two even unit cubes meet at a face and no two odd unit cubes meet at a face.

Supplementary Figure 3

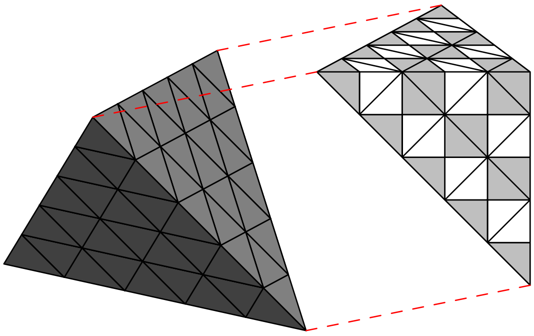

We remove tetrahedra from the cubic lattice shown in Supplementary Figure 2 to obtain the four-sided tetrahedral structure we require of the gauge color code lattice. The figure shows some of the tetrahedra that have been removed from the cubic lattice to the right of the Figure. The remaining tetrahedra do not define a gauge color code dual lattice without replacing some of the removed tetrahedra, as we explain in Supplementary Note 1 and show in Supplementary Figure 4.

Supplementary Figure 4

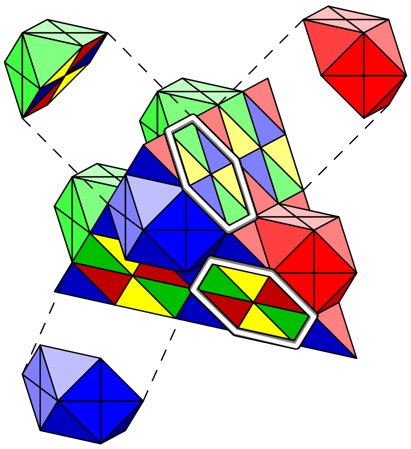

We modify the lattice of Supplementary Figure 3 to uniformly color the boundaries by replacing some of the tetrahedra that were removed from the cubic lattice. Patches of six incorrectly colored faces are outlined with white hexagons. We add additional tetrahedra to these incorrectly colored patches such that all four boundaries are uniformly colored. The Figure shows some hexagons where the additional tetrahedra are already added.

Supplementary Figure 5

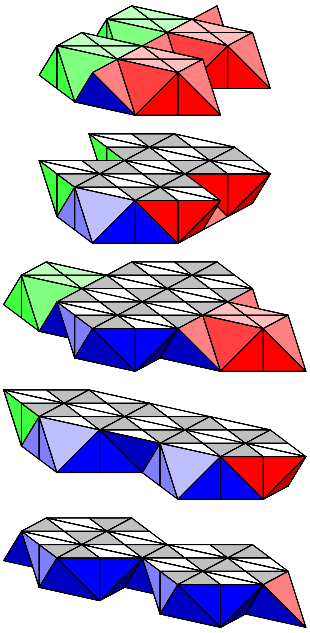

The $L = 5$ lattice where the layers of tetrahedra are separated. Interior tetrahedra are colored white and gray.

Supplementary Figure 6

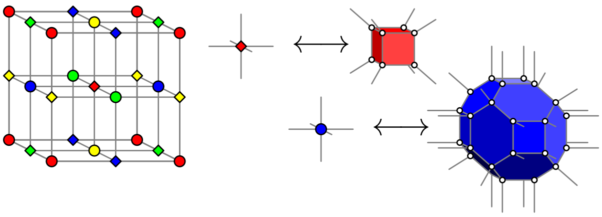

A repeating cell of the lattice that consists of eight of the fundamental unit cubes. Stabilizers of weight eight and thirty-two are represented by diamonds and circles on the vertices of the repeating cell respectively, as shown in the key at the right of the Figure. Opposite faces of the repeating cell are equivalent.

Supplementary Figure 7

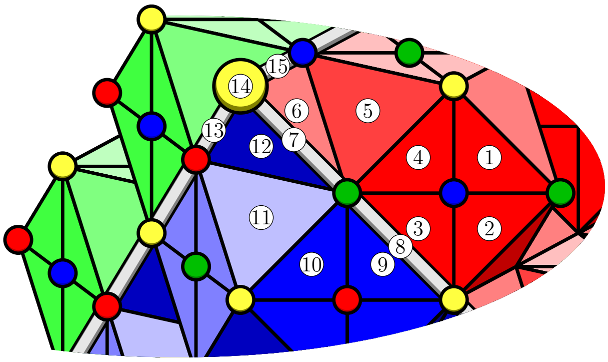

Qubits on the exterior of the gauge color code lattice. Qubits lie on all the faces, on the edges where two differently colored boundaries meet, fattened and colored in gray in the figure, and on the vertex where three differently colored boundaries meet, marked by a large yellow vertex in the Figure. Qubits are numbered in accordance with Supplementary Note 2. Qubits 7, 8, 13 and 15 lie on lattice edges and qubit 14 lies on the vertex where the red blue and green vertices meet. All other labeled qubits lie on the external faces of the lattice.

Supplementary Figure 8

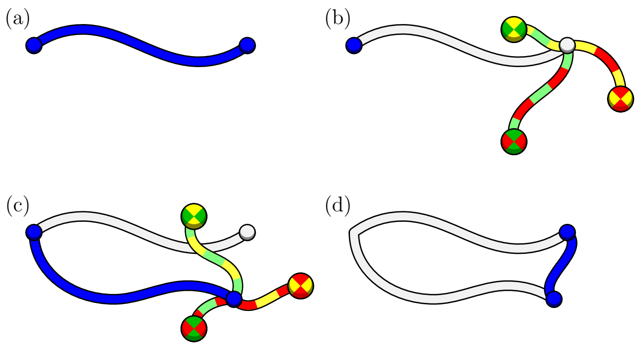

Errors introduced during fault-tolerant error correction. (a) An error string that, given perfect measurement outcomes, generates two blue stabilizer defects. (b) Measurement errors occur when measuring face operators. The measurement errors are marked as strings on the dual lattice. Gauge defects are identified where strings of measurement errors terminate. The original physical error is marked in gray. (c) During syndrome estimation, we incorrectly estimate the position of the measurement errors, and thus incorrectly identify the true position of the stabilizer defect. The correction operator we apply is represented by a blue string. (d) The correction operator we apply to correct the estimated stabilzer defect introduces a new error to the system. The net effect of the initial physical error and the correction operator is effectively the discrepancy in the estimate of the stabilizer defect position, as is marked by the blue error string.

SUPPLEMENTARY NOTES

Supplementary Note 1

Here we elaborate on the dual lattice we use to construct the gauge color code simulated in the main text. The gauge color code dual lattice is a neatly stacked structure of many tetrahedra. Conveniently though, the lattice dual to the lattice geometry shown in Figure 1(a) in the main text is composed of odd and even cubic units of five tetrahedra, shown in white and gray, respectively, in Supplementary Figure 1(a). We also show the decomposition of an even unit cell in Supplementary Figure 1(b). An odd unit cell differs from an even unit cell only by a $\pi/2$ rotation about any of the canonical axes. Unit cubes on the boundaries of the lattice are modified simply by removing subsets of their tetrahedra.

To ease the explanation of the lattice construction, it is instructive to begin with a cubic block of fundamental cubes, as shown in Supplementary Figure 2. The block must have an odd linear dimension of unit cubes. Tetrahedra are removed from this block to find an appropriate four-sided structure, and finally some of the tetrahedra that have been removed are once again replaced to find suitable boundaries that can be correctly colored.

In the dual representation we require that the vertices of the lattice are four colorable, i.e. we can consistently color every vertex with one of four colors, red, green, yellow or blue, such that no two vertices of the lattice that share an edge can have the same color. We show a single tetrahedra whose vertices are correctly colored in Supplementary Figure 1(c). The vertices of the dual lattice in Supplementary Figure 2 are indeed four colorable.

The gauge color code requires four distinct boundaries where boundaries differ by the subset of colors of the vertices that lie on their surface. Specifically, a boundary of a given color contains no vertices of that color, i.e. a green boundary contains only red, blue and yellow vertices. We therefore look for a tetrahedral structure with four boundaries of four different colors. We design the correct structure by first removing large subsets of fundamental tetrahedra to find the global shape of the lattice. We find a four-sided tetrahedral structure by removing four corners of the cubic block of unit cubes, as shown in Supplementary Figure 3.

The lattice shown in Supplementary Figure 3 does not define the gauge color code. This is because we cannot four-color the lattice such that all four boundaries of the lattice are correctly three colored, as we have explained. However, by replacing some of the tetrahedra we have removed from the lattice, we recover suitable boundaries. To find three-colored boundaries it is convenient to color the faces of the fundamental tetrahedra. We assign to each face of the tetrahedra the color that is not given to any of its adjacent vertices, as shown in Supplementary Figure 1(c).

With this face coloring, we require that the lattice has four distinct, uniformly colored boundaries, as shown in the main text in Figure 1(b).

We modify the tetrahedral lattice shown in Supplementary Figure 3 to find a suitable lattice. In Supplementary Figure 4 we color the external faces of the fundamental tetrahedra to show the additional tetrahedra we must replace on the surface of the sheered lattice in Supplementary Figure 3. In particular, we observe hexagonal 'patches' of faces which are inconsistently colored compared with the rest of the boundary. We outline some of these hexagonal patches in white. At the center of each of these hexagons lies a single vertex of a color that is not suitable for the given boundary. To rectify this we replace all of the removed tetrahedra to the lattice that were originally touching the central vertex of each hexagonal patch. Upon doing so we recover the dual lattice shown in the main text. In Supplementary Figure 4 we show some of the 'bulbs' of sixteen tetrahedra we reintroduce to the lattice. Some of the bulbs have already been reattached in the Figure. For the convenience of the reader we show the lattice separated into layers in Supplementary Figure 5.

We finally remark that the lattice we consider is particularly convenient for simulation because the stabilizers lie on the vertices of a square lattice. In Supplementary Figure 6 we show a repeating unit of the lattice geometry on a cubic lattice. In the Figure stabilizers are represented by circular and diamond-shaped vertices of appropriate color as explained by the key.

Supplementary Note 2

Here we analyse the lattice by counting the total number of qubits as a function of L. We also count the number of stabilizers, and the number of gauge operators. We also discuss the qubit support of the stabilizers, and the gauge operators. The quantities evaluated in this Section serve as good sanity checks for readers that are reproducing the gauge color code lattice.

Qubits

The gauge color code is a complicated system where qubits are placed on tetrahedra, and on subsets of faces, edges and vertices of the dual lattice we described in the previous Section. A qubit is placed on each of the exterior vertices where three differently colored boundaries meet. We therefore have vertex qubits

$$Q_v(L) = 4.$$

A qubit is also placed on exterior edges of the lattice where two differently colored boundaries meet. We have

$$Q_e(L) = 6L,$$

qubits on exterior edges of the lattice.

The lattice has qubits placed on the exterior faces of the dual lattice. We find that there are

$$Q_f(L) = 9L^2 - 5,$$

face qubits.

Finally, the lattice has a qubit on each of its tetrahedra. We find

$$Q_t(L) = (5L^3 + 24L^2 - 2L - 24)/3,$$

tetrahedron qubits.

A gauge color code following our construction therefore has $Q(L) = Q_v(L) + Q_e(L) + Q_f(L) + Q_t(L)$ qubits, which is explicitly

$$Q(L) = (5L^3 + 51L^2 + 16L - 27)/3,$$

qubits.

Stabilizers in the Dual Lattice

Each vertex of the lattice supports two stabilizers. We find the number of vertices

$$v(L) = (L^3 + 12L^2 + 5L - 6)/3.$$

Each stabilizer represented by a given vertex acts on every vertex, edge, face or tetrahedron qubit that contains the respective stabilizer vertex. To make this statement more rigorous, we can specify each object on the lattice that supports a qubit by a list of vertices, v, that are contained by the respective object. A vertex qubit is specified by a single vertex, $v = \{v\}$, an edge qubit is specified by a pair of vertices, $e = \{v_1, v_2\}$, each triangular face that supports a qubit is specified by three vertices $f = \{v_1, v_2, v_3\}$, and a tetrahedron is specified by its four vertices $t = \{v_1, v_2, v_3, v_4\}$.

Given that the different objects of the lattice that support qubits, known as simplices, can be uniformly denoted by a lists of vertices of varying length, we are free to group all vertex qubits, edge qubits, face qubits and tetrahedron qubits into the set of qubits, $\mathcal{Q}$, using this simplicial description. Written as simplices, vertex, edge, face and tetrahedron qubits only differ by the length of their list of vertices. Using this notation we can conveniently write down stabilizer operators

$$S_v^X = \prod_{Q \ni v} X_Q, \quad S_v^Z = \prod_{Q \ni v} Z_Q, \tag{1}$$

where we take the product of all qubits $Q \in \mathcal{Q}$ that contain vertex v. We point out that we have used notation here that is inconsistent with the main text, as here we index stabilizers by vertices, v, whereas in the main text we chose the index c to represent cells that support stabilizers. This reflects the change from the primal to dual lattice notation.

Gauge Operators in the Dual Lattice

We finally find the number of gauge operators we use in the simulation in the main text, and explicitly describe the supports of the gauge operators on dual lattice. Gauge operators are represented on edges, and on exterior vertices of the dual lattice. Instead of counting the number of gauge operators exactly, we count distinct supports of gauge operators. The number of supports are exactly the number of faces on the primal lattice. For each support, s, we have two gauge operators, G_s^X and G_s^Z. On the dual lattice, gauge supports are uniquely represented by either an edge, e, or a pair (v, C) that contains a vertex, v, and a color $C \in \mathcal{C}$ from the set of colors defined in the main text.

To count the gauge supports, we first find the number of edges contained on the lattice, $e(L)$. We find this number using the Euler characteristic

$$\chi_{\rm 3D} = v(L) - e(L) + f(L) - t(L), \tag{2}$$

where $\chi_{\rm 3D} = 1$ for a 'ball-shaped' triangulation, such as that which describes the gauge color code, and where $v(L)$, $f(L)$ and $t(L)$ are the number of vertices, faces and tetrahedra of the lattice, respectively. We already have the number of tetrahedra $t(L) = Q_t(L)$, and $v(L)$ is already counted to find the number of stabilizers. We easily find the number of faces of the lattice $f(L)$ using the fact that each interior face lies on the surface of two tetrahedra, and each tetrahedra has four faces. Following this, up to the exterior faces, we can regard each tetrahedra as contributing half to the face count per face of each tetrahedra. We therefore obtain a contribution of $\sim 2Q_t(L)$ faces for the tetrahedra of the lattice. To account for exterior faces, we must add an additional half-unit per exterior face of the lattice to count the total number of faces, giving the number of faces $f(L) = 2Q_t(L) + Q_f(L)/2$. Explicitly, we have

$$f(L) = (20L^3 + 123L^2 - 8L - 111)/6.$$

Using $f(L)$ and Eqn. (2) we find the number of edges

$$e(L) = (4L^3 + 33L^2 + 2L - 27)/2.$$

Each edge of the lattice represents one gauge support that acts on all edge, face, and tetrahedron qubits that contain the respective edge. We use once again the simplicial notation to denote gauge supports represented by edges, e, such that

$$G_e^X = \prod_{Q \ni \{v_1, v_2\}} X_Q, \quad G_e^Z = \prod_{Q \ni \{v_1, v_2\}} Z_Q, \tag{3}$$

where we take the product over all qubits $Q \in \mathcal{Q}$ that contain both vertices of edge $e = \{v_1, v_2\}$. We point out that no vertex qubit on the lattice can contain the two vertices of an edge, and therefore vertex qubits are not found in gauge operator supports associated to edges.

Gauge supports are also represented by vertices v on the exterior of the lattice. Each exterior vertex represents either one, two or three gauge supports. For exterior vertices that represent multiple gauge operator supports, we specify the supports uniquely with a vertex, v, and a color $C \in \mathcal{C}$.

Specifically, a gauge support of a given exterior vertex v contains a *subset* of the vertex, edge or face qubits that contain v. The subset of lattice objects $Q \ni v$ are taken to be all of those that do not contain any vertices of one particular color.

To make this statement rigorous using the simplex notation, we define the function $C : v \to \mathcal{C}$ that returns the color of the vertex as defined by the choice of the four-coloring of the lattice.

Rigorously, external vertex gauge operators are written

$$G^X_{(v,C)} = \prod_{Q \ni v \backslash C} X_Q, \quad G^Z_{(v,C)} = \prod_{Q \ni v \backslash C} Z_Q, \tag{4}$$

where we use a shorthand notation '$Q \ni v\backslash C$' to denote qubits Q that contain vertex v, but do not contain any vertices of color $C \in \mathcal{C}$, i.e. where $C(v') \neq C$ for all $v' \in Q$. We point out that all tetrahedra contain one vertex of each color, and as such, no tetrahedra appear in the gauge supports represented by exterior vertices.

In some cases there are exterior vertices with multiple nonempty subsets $Q \ni v\backslash C$ for different choices of C. In what follows we explicitly consider exterior vertices that contain one, two, and three gauge operator supports. We will see that exterior vertices in the middle of a boundary will contain only one gauge operator support, exterior vertices found where two different colored boundaries meet contain two distinct gauge operator supports, and vertices that lie where three different boundaries meet contain three different gauge operator supports.

Exterior vertices in the middle of a boundary, far away from any edge or vertex qubits, denote one gauge operator support. We explicitly consider the example of the gauge support associated to the blue vertex shown on the red boundary in Supplementary Figure 7. The gauge operator support associated to this vertex act on the qubits labeled 1, 2, 3 and 4. All qubits that contain the exterior vertex of interest contain a yellow, a green, and a blue vertex, but no red vertex.

Vertices that lie at a point where two differently colored boundaries meet represent two distinct gauge operator supports. We consider the the green vertex in Supplementary Figure 7 that lies where the blue boundary and the red boundary meet. One of the supports acts on the qubits labeled 3, 4, 5, 6, 7 and 8. Common to all of these qubits is that none of their faces or edges contain a red vertex. The other support acts on qubits 7, 8, 9, 10, 11 and 12. Different from the first discussed support associated to the green vertex, none of the faces or edges involved in this support contain a blue vertex.

We finally consider the support of the gauge operators associated to the vertices that lie where three boundaries meet, such as at the large yellow vertex shown in Supplementary Figure 7 that contains the qubit indexed 14. This vertex denotes three supports, two of which are shown in the diagram. One support acts on qubits 6, 7, 14 and 15, the qubits that contain the yellow vertex of interest, but do not contain a red vertex. The other support acts on the qubits numbered 7, 12, 13 and 14, the lattice objects that contain the large yellow vertex, but do not contain any blue vertices. The third support acts on a green face qubit that cannot be seen in the diagram.

We can now count the number of gauge supports we must measure to realize fault-tolerant error correction using the gauge color code. We count the number of exterior vertices $v^E(L)$ with the expression

$$v^E(L) = (9L^2 - 1)/2.$$

Then, using that the $6(L-1)$ vertices that lie where two boundaries meet represent two gauge operator supports, and four vertices that lie where three boundaries meet represent three gauge operator supports, we find the number of gauge operator supports $G(L) = e(L) + v^E(L) + 6(L-1) + 8$, giving the number of gauge operator supports

$$G(L) = 2L^3 + 21L^2 + 7L - 12.$$

Twice this quantity gives the number of gauge operators we would use to perform fault-tolerant error correction with the gauge color code, if we wish to identify both Pauli-X and Pauli-Z type errors.

Supplementary Note 3

In the main text we identify the threshold error rate as a function of the number of rounds of error correction that are performed, N, to show that the threshold error rate is robust in the limit $N \rightarrow \infty$. It is important to look at the single-shot error-correction scheme after repeated applications because, when one considers noisy measurements, the error-correction protocol will leave some residual noise on the code. In general, this residual noise is not easily characterized. In fact, the noise introduced to the system is a complex function of the physical error rate, the measurement error rate, and the choice of error correction protocol. Given that the nature of the residual noise is not well understood, it is not clear that the error-correction protocol will be able to successfully deal with the residual noise after many cycles of error correction.

In Supplementary Figure 8 we give an example of a mechanism that leads to the development of a correlated error. We show a physical error in Supplementary Figure 8(a). The error is represented by a string. Given perfect measurements, we expect to observe two stabilizer defects at either end of the error string, the left and right stabilizer defect.

For this example, some measurement errors occur in the vicinity of the right stabilizer defect when we attempted to learn the positions of stabilizer defects. The measurement errors, where face operators return incorrect measurement outcomes, are represented by multi-colored strings in Supplementary Figure 8(b). Gauge defects lie at the points where the strings of measurement errors terminate. Given these measurement errors occur, we cannot be sure of the true location of the right stabilizer defect. Instead, we can make use of the positions of the three gauge defects, and knowledge of the noise model, to attempt to determine the location of the stabilizer defect.

Our ability to predict the positions of stabilizer defects depends on our choice of syndrome estimation algorithm. In Supplementary Figure 8(c) we show where the syndrome estimation algorithm mistakenly predicts measurement errors. The measurement errors that have been predicted are represented by strings that terminate at the gauge defects, and branch at the estimated stabilizer defect. The incorrect estimation made by the algorithm leads us to believe that the stabilizer defect is not in its true location, but is displaced to some estimated location.

Next, we apply a correction operator to attempt to repair the initial physical error drawn in Supplementary Figure 8(a). However, using the estimated position of the right stabilizer defect, we apply a correction operator that connects the left stabilizer defect to the estimated right stabilizer defect, as shown by a blue string in Supplementary Figure 8(c), thus introducing additional errors to the code. The initial error, and the applied correction operator is shown in gray in Supplementary Figure 8(d).

Due to the topological nature of the string errors in the gauge color code, the net effect of the initial error and the correction operator equates to the discrepancy between the true position of the right stabilizer defect, and the position of the estimated stabilizer defect. The effective error is shown by the blue error string in Supplementary Figure 8(d). This is easily checked as we can continuously deform the gray string of the Figure onto the blue string. In other words, in the gauge color code the gray error string is equivalent to the blue error string up to multiplication by gauge operators.

In general it is not clear how able a syndrome estimation algorithm is for correctly predicting the positions of stabilizer defects. A bad syndrome estimation algorithm might displace stabilizer defects over long distances compared to their true positions, in which case large correlated errors that we cannot correct may develop. Moreover, it is not clear that the character of the residual noise remains constant over many error-correction cycles. Indeed, one should be concerned that an error correction protocol might cause correlations to develop over repeated use while information is stored. It is therefore important to study a single-shot error-correction protocol over many cycles of error correction to interrogate its performance, and to check that the noise incident to a code achieves a steady-state in the long-time limit. In doing so, we are able to establish that a code has a finite threshold after an arbitrarily long time.
